# Hydrogen Molecule Delivery System to Ischemic Intestine Using Resuscitative Endovascular Balloon Occlusion of Aorta in Hemorrhagic Shock—A Proof-of-Concept Study

**DOI:** 10.3390/biomedicines14020455

**Published:** 2026-02-18

**Authors:** Takahiro Yamanaka, Tadashi Matsuoka, Koichiro Homma, Tomoyoshi Tamura, Sayuri Suzuki, Shohei Suzuki, Daiki Kaito, Jo Yoshizawa, Keitaro Yajima, Soichiro Ono, Katsuya Maeshima, Eiji Kobayashi, Motoaki Sano, Junichi Sasaki

**Affiliations:** 1 Department of Emergency and Critical Care Medicine, Keio University School of Medicine, Shinjuku-ku 160-8582, Japan; shianzhon@gmail.com (T.Y.); tadashi_matsuoka1984@yahoo.co.jp (T.M.);; 2 The Center for Molecular Hydrogen Medicine, Keio University Global Research Institute, Shinjuku-ku 160-8582, Japan; 3 Department of Kidney Regenerative Medicine, Jikei University School of Medicine, Minato-ku 105-8461, Japan; 4 Department of Medicine and Clinical Science, Yamaguchi University Graduate School of Medicine, Ube 755-8505, Japan

**Keywords:** hemorrhagic shock, resuscitative endovascular balloon occlusion of the aorta, hydrogen

## Abstract

**Background**: The use of resuscitative endovascular balloon occlusion of the aorta (REBOA) for hemorrhagic shock in the torso has become increasingly common as a bridge to definitive hemostasis. Hydrogen molecules, distributed throughout the bloodstream, alleviate ischemic injury but cannot reach ischemic organs during REBOA use. This study investigates whether intra-aortic irrigation with hydrogen-dissolved saline under REBOA use delivers hydrogen to the intestine in a swine hemorrhagic shock model. **Methods**: We induced volume-regulated hemorrhagic shock in a 40 kg female swine. Following this, hydrogen-dissolved saline irrigation was initiated through an intra-aortic catheter positioned distal to the REBOA balloon. Hydrogen concentration in the portal vein was determined in four models: controlled hemorrhagic shock with full REBOA inflation during the standard occlusion time, uncontrolled hemorrhagic shock with liver injury and full REBOA inflation during the extended occlusion time, uncontrolled hemorrhagic shock with liver injury and partial REBOA inflation during the extended occlusion time, and as the control model, controlled hemorrhagic shock with full REBOA inflation during the standard occlusion time with normal saline irrigation without hydrogen. **Results**: Hydrogen concentration in the portal vein was found to be 0.224 mg/L (13.998%) in the controlled hemorrhagic shock model with full REBOA inflation, 0.049 mg/L (3.063%) in the uncontrolled hemorrhagic shock model with liver injury and full REBOA inflation, 0.018 mg/L (1.125%) in the uncontrolled hemorrhagic shock model with liver injury and partial REBOA inflation, and 0.002 mg/L (0.015%) in the control model. These results demonstrate the presence of hydrogen in the portal vein under different REBOA applications. **Conclusions**: Increased hydrogen concentration in the portal vein indicated that hydrogen was delivered to the intestine. These findings suggest an approach for drug administration during REBOA use. However, further investigations are required to establish its application in clinical settings.

## 1. Introduction

Hemorrhagic shock is a significant cause of early mortality in patients [1]. In recent years, the use of resuscitative endovascular balloon occlusion of the aorta (REBOA) for hemorrhagic shock in the torso has become more common as a bridge therapy for definitive hemostasis. REBOA can temporarily control hemorrhage and preserve blood flow to the brain and coronary arteries [2,3,4]. This procedure is relatively easier than aortic cross-clamping; therefore, REBOA is expected to be used for various diseases, including non-traumatic conditions such as ruptured aortic aneurysm, gastrointestinal bleeding, and postpartum hemorrhage [5,6,7]. However, REBOA has several disadvantages. Blocking the aorta with REBOA inhibits blood flow downstream to the visceral organs, such as the intestine, in exchange for maintaining cerebral and coronary blood flow [7]. Given the risk of ischemic injury to visceral organs, there is a time limit for keeping the aorta blocked with REBOA [8]. Various approaches, including partial inflation and intermittent deflation, have been proposed to mitigate ischemic injury [9]. However, this technique is relatively complex and lacks a uniform approach. In addition, beyond refinements in balloon management aimed at preserving distal perfusion—such as partial or intermittent REBOA inflation—there remains a need for adjunctive strategies that can be delivered to ischemic visceral organs during aortic occlusion to mitigate ischemia–reperfusion injury [9,10,11].

Molecular hydrogen (H_2_) has been proposed as a selective antioxidant and cytoprotectant in ischemia–reperfusion injury and inflammation, and a broad body of preclinical and clinical literature has explored diverse delivery routes, including inhalation, intravenous/enteral hydrogen-rich solutions, nanoparticle-based carriers, and hydrogen-enriched organ perfusates across the heart, brain, kidney, liver, and intestine [12,13,14,15,16,17,18,19,20,21,22]. However, most hydrogen delivery strategies rely on intact systemic or splanchnic perfusion, and whether adequate H_2_ reaches the distal visceral circulation during complete aortic occlusion is uncertain; notably, recent experimental work suggests that hydrogen gas inhalation after REBOA may not sufficiently attenuate REBOA-associated ischemia–reperfusion injury [19].

To address the delivery barrier created by aortic occlusion, we explored whether H_2_ could be delivered to the distal visceral circulation during REBOA by irrigating H_2_-dissolved saline into the aorta distal to the occlusion balloon. In this proof-of-concept swine study, we quantified portal venous H_2_ concentrations under clinically relevant REBOA scenarios, including a normal saline irrigation control, to assess the feasibility and pharmacokinetic detectability of this downstream intra-aortic approach. This study was not designed to evaluate therapeutic efficacy or tissue protection.

## 2. Materials and Methods

### 2.1. Overview

This study was approved by the Research Council and Animal Care and Use Committee of the Research Institute of Keio University (20005-(2)). This study included four female swine (39–42 kg; Zen-noh Premium Pigs, Zen-noh Livestock, Tokyo, Japan), which were maintained in individual stainless-steel cages in a temperature- and light-controlled room (20 °C, 12 h light-dark cycle). The animals were specially bred, socialized, vaccinated, free from common domestic swine diseases, and had free access to food and water prior to the experiment. We complied with the ARRIVE guidelines.

### 2.2. Study Design

The study employed a translational swine model to determine the possibility of drug delivery under the REBOA use as a proof of concept study. The objective of this study was to demonstrate whether drugs with an anti-ischemic effect, i.e., H_2_ gas, could reach the intestine under REBOA use. Herein, we investigated the concentration of H_2_ gas molecules in the portal vein (PV), the outflow from the intestine, under REBOA use in different hemorrhagic shock models with/without solid organ injury. The presence of H_2_ molecules would indicate that H_2_ can potentially reach the intestine through intra-aortic irrigation with H_2_-dissolved saline under REBOA use.

### 2.3. Animal Preparation

**Figure 1:**
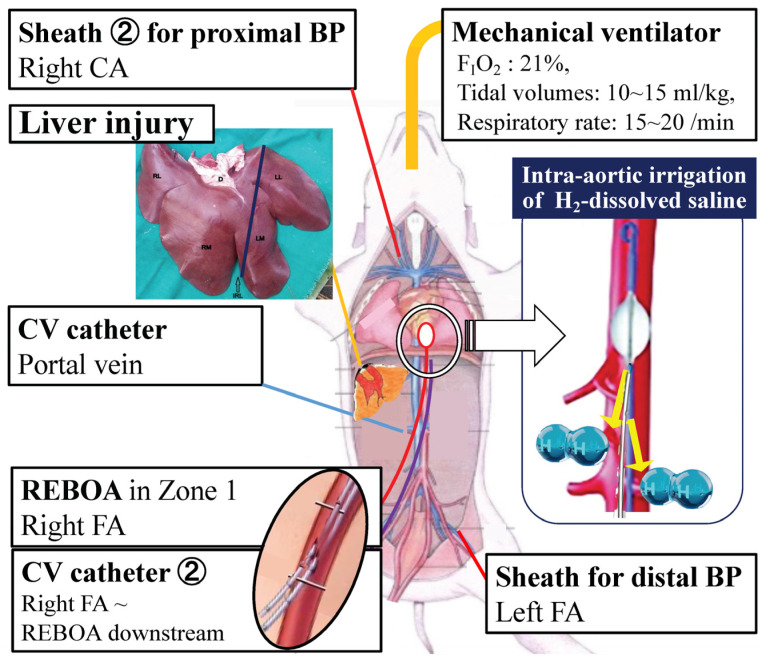
Experimental setting. An intra-aortic catheter to irrigate the H_2_-containing saline was positioned distal to the REBOA balloon in zone 1. The H_2_-dissolved saline was administered to the aorta downstream of the REBOA blockage through a CV catheter. The H_2_ concentration produced in this experiment was 2.2 mg/L. BP, blood pressure; CA, carotid artery; CV, central vein; FA, femoral artery; REBOA, resuscitative endovascular balloon occlusion of the aorta.

Animals were premedicated with an intramuscular mixture of midazolam (0.2 mg/kg), medetomidine (20 μg/kg), and butorphanol (0.2 mg/kg). Following isoflurane induction and endotracheal intubation, maintenance anesthesia consisted of 2% isoflurane. The animals were mechanically ventilated in a volume-controlled mode (FIO_2,_ 21%; tidal volume, 10–15 mL/kg; respiratory rate, 15–20 breaths per min) and placed on a warming blanket set at 37 °C to minimize hypothermia. The right carotid artery and the bilateral femoral arteries were exposed. Arterial access was obtained for blood collection and hemodynamic monitoring. Proximal aortic pressure was measured via a 5 Fr introducer sheath (Super Sheath; Togo Medikit Corporation, Miyazaki, Japan) inserted into the right common carotid artery. Distal aortic pressure was similarly obtained via a 5 Fr introducer sheath (Super Sheath) inserted in a retrograde manner into the left femoral artery. With laparotomy, a central venous catheter (14G CV catheter kit single lumen; Cardinal Health, Tokyo, Japan) was inserted into the PV via the pancreatic vein to collect blood samples (Figure 1).

### 2.4. REBOA Placement and H_2_ Administration Route

A 7 Fr REBOA catheter (Rescue Balloon^®^, Tokai Medical Products, Kasugai city, Japan) was retrogradely inserted via the right femoral artery. The REBOA balloon was located in zone 1, and the aorta from the left subclavian artery to the celiac artery was confirmed using extra-anatomical measurements prior to placement and through palpation at the completion of the experiment. Another central venous catheter (12G CV catheter kit double lumen; Cardinal Health, Tokyo, Japan) for irrigation with H_2_-containing saline was retrogradely inserted into the right femoral artery along with the REBOA (Figure 1). The top tip of the catheter was positioned distal to the REBOA balloon, and H_2_-containing saline was administered through the lumen of the catheter (Figure 1).

### 2.5. Dissolving H_2_ Gas into Saline

**Figure 2:**
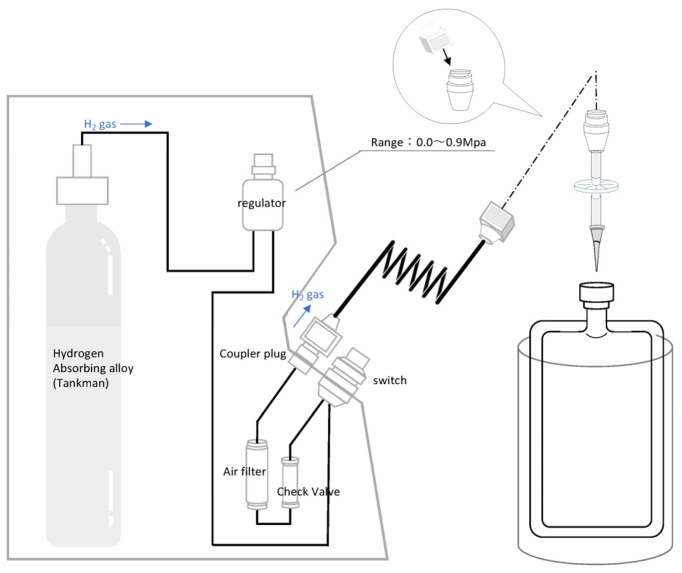
Structure of the hydrogen (H_2_) filling machine. H_2_ gas was injected into the plastic bag of normal saline at the desired pressure through connecting the outlet of the plastic bag (the outlet of which was replaced with one that integrated a coupler plug with a stop valve) to the coupler socket with a stop valve on the main body of the hydrogen gas filling system. The H_2_ released from the H_2_ storage alloy in the H_2_ gas-filling system was adjusted to the desired pressure using a regulator (pressure control device).

H_2_-dissolved saline was prepared as described previously [17]. The H_2_-absorbing alloy canister (Japan Steel Works, Ltd., Shinagawa-ku, Japan) is a cylinder containing an H_2_-absorbing alloy that reversibly absorbs and releases H_2_. Given that the internal pressure of the canister does not exceed 1 MPa, it is not deemed a high-pressure gas, as defined in the Japan High-Pressure Gas Safety Act. The H_2_ transfer device was assembled immediately before use, and H_2_ was injected into normal saline from the H_2_-absorbing alloy canister through a pressure regulator valve (Figure 2). When the gauge pressure reached 0.06 MPa, the plastic bag containing normal saline was disconnected from the pressure regulator valve. After vigorous manual shaking for 30 s, the bag containing normal saline was placed on ice and opened to the atmosphere. The H_2_ concentration produced during this experiment was 2.2 mg/L, which was >1.6 mg/L, the saturation point of H_2_ gas in water at room temperature without external pressure.

### 2.6. Experimental Model

To demonstrate the concept of drug delivery under REBOA use, we established the following models considering the application of REBOA in clinical scenarios and basic research: controlled/uncontrolled hemorrhagic shock, standard (60 min) [23,24,25] /prolonged (90 min) [9,23,24,26,27] inflation time, with/without solid organ injury, or full/partial inflation. Since hydrogen gas is naturally produced by the gut microbiota and absorbed into the PV, its concentration in the PV was evaluated in the models without hydrogen-dissolved saline irrigation. Each model was performed once (*n* = 1), and the feasibility of hydrogen delivery was evaluated in each case.

Model 1: Controlled hemorrhagic shock model with full REBOA inflation for the standard occlusion time: 60 min

Controlled hemorrhage, a weight-based 20% total blood volume-controlled hemorrhage, was induced by withdrawing a predefined volume of blood through a 5 Fr sheath within 15 min, based on previous reports describing a controlled hemorrhagic shock pig model [10]. Immediately after shock induction, the REBOA balloon was inflated for 60 min. Successful occlusion of the aorta was confirmed through the loss of distal pulses in the left lower extremity, with an increase in mean arterial pressure (MAP) in the carotid artery. While the REBOA was inflated, the H_2_-dissolved saline was delivered to the aorta downstream of the REBOA occlusion through a central venous catheter inserted along with the REBOA. After 60 min of inflation with the irrigation of the H_2_-dissolved saline, the animal was euthanized by administering a KCL injection.

Model 2: Uncontrolled hemorrhagic shock with liver injury, accompanied by REBOA inflation for an extended occlusion time: 90 min

Following the induction of controlled hemorrhage for 15 min, as described above, a standardized high-grade liver injury was established by cutting the hepatic parenchyma medially, immediately distal to the confluence of segment 4 and the left lateral hepatic vein, using large scissors [10]. The midline laparotomy was rapidly closed after removing the liver segment. The REBOA balloon was immediately inflated for 90 min, and H_2_-dissolved saline was delivered to the aorta downstream of the REBOA occlusion. After 90 min of inflation, the animal was euthanized by administering a KCL injection.

Model 3: Uncontrolled hemorrhagic shock with liver injury, accompanied by partial REBOA inflation for an extended occlusion time: 90 min

Following the induction of controlled hemorrhage within 15 min, as described above, a standardized high-grade liver injury was established. The midline laparotomy was rapidly closed after the removal of the liver segment, and the REBOA balloon was inflated for 10 min for initial clot formation and stabilization, as described previously [9]. H_2_-dissolved saline was delivered to the aorta downstream of the REBOA occlusion. After 10 min of full occlusion, the balloon was promptly deflated until the distal aortic waveform reappeared. During the experimental period, the degree of inflation was modified to maintain adequate proximal and distal blood pressures at the minimum level to confirm distal pulses in the right lower extremity [9]. After 90 min of full and partial inflation, the animal was euthanized by administering a KCL injection.

Model 4 (control model): Controlled hemorrhagic shock with full REBOA inflation and normal saline irrigation for an extended occlusion time: 60 min

A controlled hemorrhage lasting 15 min was induced as described above. Immediately after shock induction, the REBOA balloon was inflated for 60 min. Successful occlusion of the aorta was confirmed through the loss of distal pulses in the left lower extremity, with an increase in MAP in the carotid artery. During REBOA inflation, normal saline was delivered to the aorta downstream of the REBOA occlusion through a central venous catheter inserted alongside the REBOA. After 60 min of inflation with normal saline irrigation, the animal was euthanized by KCl injection.

### 2.7. Data Collection

During all experiments, physiological data, including proximal carotid blood pressure, distal femoral artery blood pressure, and heart rate, were continuously monitored and recorded using a polygraph (Powerlab System, Chart; AD Instruments, Bella Vista, NSW, Australia). To measure the H_2_ concentration, blood samples were collected from catheters inserted into the PV at baseline and every 15 min throughout the experimental period.

### 2.8. Measuring H_2_ Concentration

To measure blood H_2_ concentration, we first inserted a needle into the rubber cap of a sealed 13.5 mL vial, extracted 1 mL of air, and injected 1 mL of blood. Wax was immediately applied to the rubber cap to seal the injection port and prevent outgassing. H_2_ in the blood was released into the air phase in a sealed vial. The air in the vial into which the blood was initially injected contained almost no H_2_ gas (0.5 ppm *v*/*v*); therefore, most of the H_2_ gas moved from the blood (liquid phase) to the air (gas phase). Through examining the concentration of H_2_ gas in the air inside the vial, the concentration of H_2_ gas in the blood could be estimated. A portion of the air phase (0.2 mL, 0.4 mL, or 1 mL, depending on the H_2_ concentration) was collected from the vial, and the H_2_ concentration was measured using gas chromatography TRIlyzer mBA-3000 (Taiyo Co., Ltd., Kochi-shi, Japan). A calibration curve was constructed using standard H_2_ at concentrations of 0 (nitrogen gas), 5, 50, and 130 parts per million (ppm). Each sample was analyzed twice. Simultaneously, air was collected from the same location into a blood-free vessel, and the concentration of H_2_ gas was determined. Additionally, the H_2_ concentration in normal saline, prepared without dissolved hydrogen, was measured as the control (0.0001 ppm *w*/*w*). The value of H_2_ in the air was 0.5 ppm *v*/*v*, which exceeds the limit of quantification. Each measured value for the sample was obtained by subtracting the air values.

### 2.9. Administration Rate

Because complete REBOA markedly reduces or abolishes distal aortic flow, downstream delivery requires infusion-driven transport. This study was designed as a feasibility/pharmacokinetic proof-of-concept rather than dose optimization; therefore, we used a pragmatic dosing strategy constrained by device capability and physiologic plausibility. In full REBOA models, H_2_-dissolved saline was infused at 1.5–2.0 L/h (0.025–0.033 L/min), including the device maximum of 2.0 L/h to ensure measurable delivery under no-flow conditions. In the partial REBOA model, where some native distal flow is preserved, the infusion rate was reduced to 0.1 L/h to approximate a clinically feasible adjunct. For contextual interpretation, we used a reference portal venous concentration of 0.016 mg/L (approximately 1% saturation) based on prior swine pharmacokinetic data following enteral hydrogen administration [20].

## 3. Results

### 3.1. Model 1: Controlled Hemorrhagic Shock Model with Full REBOA Inflation for the Standard Occlusion Time: 60 Min

#### 3.1.1. Hemodynamics During REBOA Application

**Figure 3:**
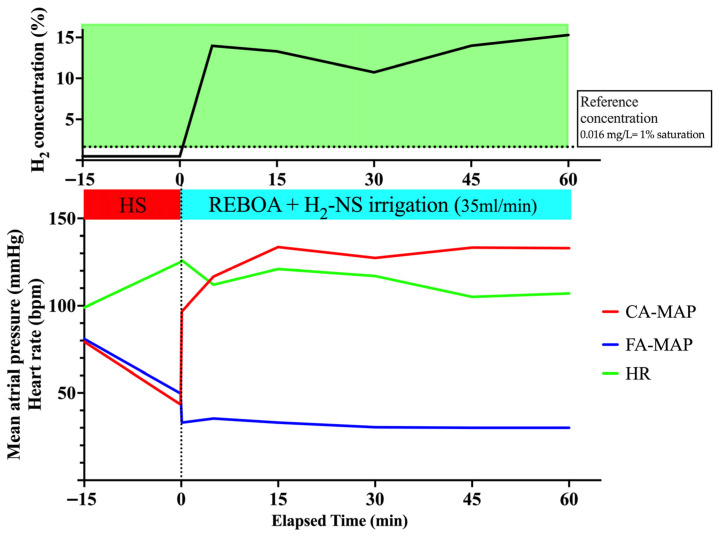
Changes in hemodynamic parameters and H_2_ concentration with normal time full-inflation and controlled hemorrhagic shock without liver injury (*n* = 1). The horizontal dotted line indicates the reference concentration (0.016 mg/L; ~1% saturation). CA, carotid artery; FA, femoral artery; H_2,_ hydrogen; HR, heart rate; HS, hemorrhagic shock; NS, normal saline; MAP, mean arterial pressure; REBOA, resuscitative endovascular balloon occlusion of the aorta.

The mean carotid and femoral blood pressures and heart rate are shown in Figure 3. Shock induction by removing a predefined amount of blood reduced the MAP in the carotid and femoral arteries to ~40 mmHg. After the initial full inflation of the REBOA, the MAP in the carotid artery immediately increased, whereas the pulse in the femoral artery decreased, indicating that occlusion was achieved using REBOA. In Experiment 1, the MAP in the carotid artery was maintained at an elevated level of ~130 mmHg during the experimental period, thereby indicating that the use of the REBOA with full inflation for hemorrhagic shock was successful.

#### 3.1.2. H_2_ Concentration in the PV

Changes in H_2_ concentration are shown in Table 1 and Figure 3. Irrigation with H_2_-dissolved saline immediately increased the H_2_ concentration in the PV, peaking at 0.224 mg/L (13.998%) 5 min after irrigation, and was maintained throughout the experiment. These data demonstrate that downstream irrigation during full REBOA can increase portal venous H_2_ above a reference concentration (0.016 mg/L; ~1% saturation) [20], supporting feasibility of distal delivery under complete occlusion.

### 3.2. Model 2: Uncontrolled Hemorrhagic Shock with Liver Injury, Accompanied by Full REBOA Inflation for an Extended Occlusion Time: 90 Min

#### 3.2.1. Hemodynamics During REBOA Application

**Figure 4:**
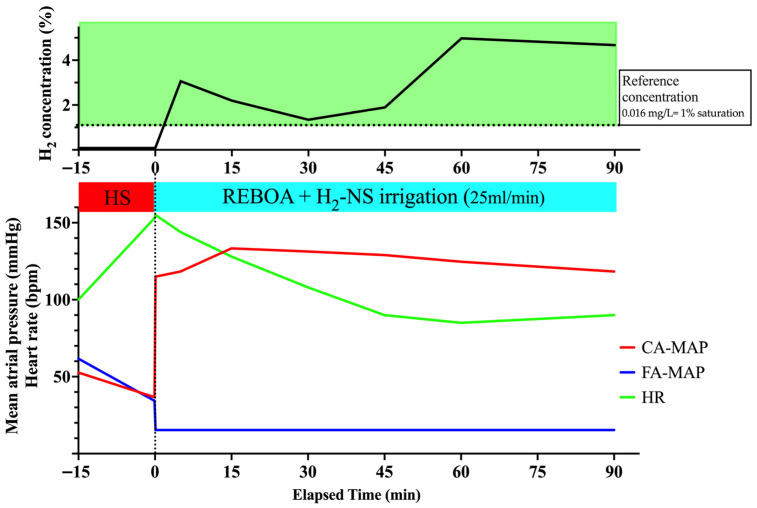
Changes in hemodynamic parameters and H_2_ concentration with extended time full-inflation and uncontrolled hemorrhagic shock with liver injury (*n* = 1). The horizontal dotted line indicates the reference concentration (0.016 mg/L; ~1% saturation). CA, carotid artery; FA, femoral artery; H_2,_ hydrogen; HR, heart rate; HS, hemorrhagic shock; NS, normal saline; MAP, mean arterial pressure; REBOA, resuscitative endovascular balloon occlusion of the aorta.

The mean carotid and femoral blood pressures and heart rate are shown in Figure 4. Induction of shock by removing a predefined amount of blood, followed by liver resection, decreased the MAP in the carotid and femoral arteries to ~40 mmHg. After the initial full inflation of the REBOA, the MAP in the carotid artery immediately increased, whereas the pulse in the femoral artery decreased. The carotid artery MAP was elevated even during uncontrolled hemorrhagic shock owing to liver resection during the extended 90 min occlusion, indicating the successful use of REBOA. The intraperitoneal hemorrhage volume was 580 mL at the end of the study period.

#### 3.2.2. H_2_ Concentration in the PV

Table 1 and Figure 4 show changes in H_2_ concentration. As observed in Experiment 1, the H_2_ concentration in the PV immediately increased and reached a peak level of 0.049 mg/L (3.063%). These data demonstrate that downstream irrigation during prolonged full REBOA can increase portal venous H_2_ above a reference concentration (0.016 mg/L; ~1% saturation) [20] even during uncontrolled hemorrhagic shock with liver injury.

### 3.3. Model 3: Uncontrolled Hemorrhagic Shock with Liver Injury, Accompanied by Partial REBOA Inflation for an Extended Occlusion Time: 90 Min

#### 3.3.1. Hemodynamics During REBOA Application

**Figure 5:**
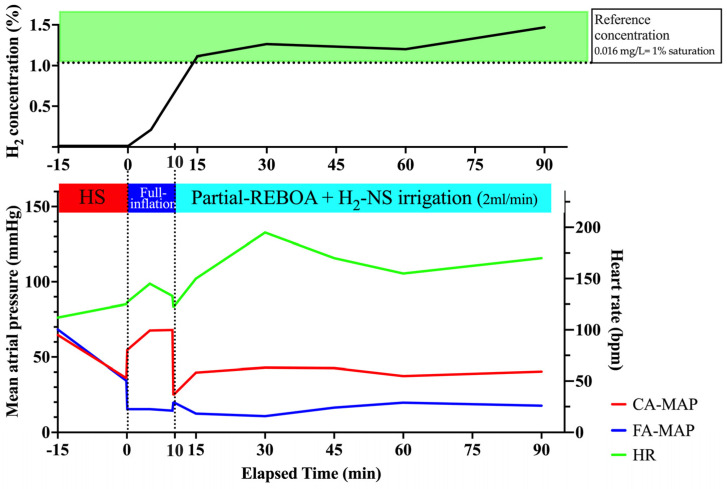
Changes in hemodynamic parameters and H_2_ concentration with extended time partial-inflation and uncontrolled hemorrhagic shock with liver injury (*n* = 1). The horizontal dotted line indicates the reference concentration (0.016 mg/L; ~1% saturation). CA, carotid artery; FA, femoral artery; H_2,_ hydrogen; HR, heart rate; HS, hemorrhagic shock; NS, normal saline; MAP, mean arterial pressure; REBOA, resuscitative endovascular balloon occlusion of the aorta.

Figure 5 presents the mean carotid and femoral blood pressures and heart rate. Induction of shock by removing a predefined amount of blood, followed by liver resection, decreased the MAP in the carotid and femoral arteries to ~40 mmHg. After the initial full inflation of the REBOA, the MAP in the carotid artery immediately increased, whereas the pulse in the femoral artery decreased. Initiating partial inflation after 10 min of full inflation reduced the MAP in the carotid artery, although it was maintained at ~40 mmHg. Simultaneously, the MAP in the femoral artery increased by ~20 mmHg, indicating that partial REBOA was successful. The intraperitoneal hemorrhage volume was 800 mL at the end of the study period.

#### 3.3.2. H_2_ Concentration in the PV

Changes in H_2_ concentration are shown in Table 1 and Figure 5. Because the irrigation rate was relatively slow during the initial full inflation period, the H_2_ concentration was not markedly elevated early; however, at the start of partial inflation, the H_2_ concentration in the PV increased to 0.0178 mg/L (1.125%) and was maintained at this level. These data demonstrate that downstream irrigation can achieve portal venous H_2_ at approximately the reference concentration (0.016 mg/L; ~1% saturation) [20] during partial REBOA in uncontrolled hemorrhagic shock with liver injury.

### 3.4. Model 4 (Control Model): Controlled Hemorrhagic Shock with Full REBOA Inflation and Normal Saline Irrigation for an Extended Occlusion Time: 60 Min

#### 3.4.1. Hemodynamics During REBOA Application

**Figure 6:**
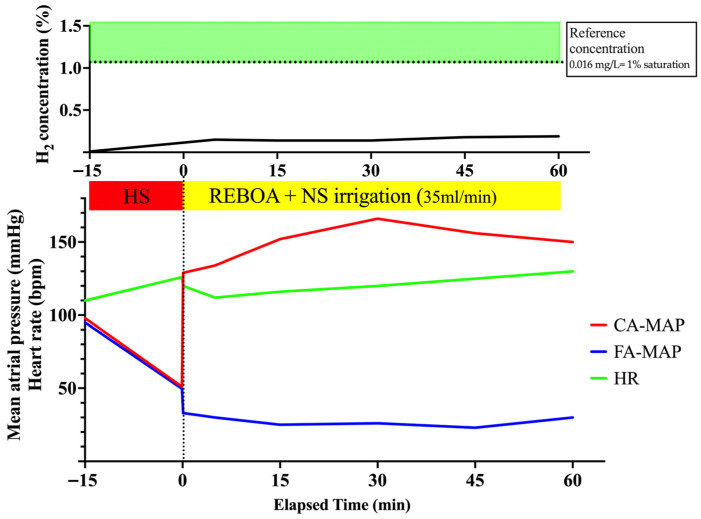
Changes in hemodynamic parameters and H_2_ concentration with normal time full-inflation with the irrigation of normal saline and controlled hemorrhagic shock (*n* = 1). The horizontal dotted line indicates the reference concentration (0.016 mg/L; ~1% saturation). CA, carotid artery; FA, femoral artery; H_2,_ hydrogen; HR, heart rate; HS, hemorrhagic shock; NS, normal saline; MAP, mean arterial pressure; REBOA, resuscitative endovascular balloon occlusion of the aorta.

The mean carotid and femoral blood pressures and heart rate are presented in Figure 6. Shock induction by removing a predefined amount of blood reduced the MAP in the carotid and femoral arteries to approximately 50 mmHg. Following the initial full inflation of the REBOA, the MAP in the carotid artery immediately increased, whereas the pulse in the femoral artery decreased, indicating that occlusion was achieved using REBOA. In Experiment 4, the MAP in the carotid artery was maintained at an elevated level of approximately 160 mmHg during the experimental period, thereby indicating that the use of the REBOA with full inflation for hemorrhagic shock was successful.

#### 3.4.2. H_2_ Concentration in the PV

Changes in H_2_ concentration are shown in Table 1 and Figure 6. Because normal saline (without dissolved H_2_) was irrigated in this model, the H_2_ concentration in the PV remained low (≤0.003 mg/L; ≤0.188%), well below the reference concentration (0.016 mg/L; ~1% saturation) [20]. This low-level signal is consistent with baseline H_2_ produced by gut microbiota and absorbed into the portal circulation.

## 4. Discussion

In the current study, we identified the presence of H_2_ molecules in the PV under the use of REBOA with downstream H_2_-dissolved saline irrigation. This finding indicates that H_2_ molecules from H_2_-dissolved saline were sufficiently delivered to the intestines of pigs with hemorrhagic shock, thereby achieving the objective of a proof-of-concept study. Moreover, this finding could lead to a novel administration route under REBOA use, potentially facilitating the delivery of anti-ischemic drugs to the intestine and liver, organs well-known to incur ischemic injury under REBOA with hemorrhagic shock. These results could lead to a novel route under REBOA use, including full or partial balloon inflation and standard or extended inflation time, potentially facilitating delivery of anti-ischemic drugs or cell-based therapies to the intestine and liver [28,29,30].

Under complete occlusion, aortic blood flow downstream of REBOA is absent. Therefore, a high administration rate is required to deliver the dissolved H_2_ saline to downstream organs. Because this study was designed as a proof-of-concept technical feasibility assessment (not a dose-finding or pharmacodynamic study), we used a pragmatic stepwise approach. We first selected the maximum flow rate permitted by our irrigation setup (2.0 L/h) to maximize the likelihood of detecting a change in portal venous H_2_ during complete occlusion. After confirming measurable portal venous H_2_ at this rate, we reduced the irrigation rate in the liver-injury full-occlusion model to decrease infused volume while maintaining detectability. In the partial-REBOA model, given expected residual distal perfusion, we further reduced the rate (0.1 L/h) to explore feasibility under substantially lower infusion volumes. These rates were chosen for signal detection and feasibility and should not be interpreted as optimal or clinically recommended. Although hydrogen gas is naturally produced by gut microbiota and absorbed into the PV, naturalist baseline concentration in the PV was quite low, as observed in Model 4 (control). These findings suggest that our novel drug delivery system delivered hydrogen to the intestine; however, we did not evaluate histological damage assessment, which needed further efficacy investigation. Given that this is a proof-of-concept study, these findings could facilitate the determination of an appropriate administration rate under the REBOA downstream irrigation of drugs.

We have previously reported that H_2_ gas inhalation mitigates ischemic injury and delays progression to irreversible shock during severe hemorrhage [15]. However, since H_2_ molecules from inhaled gas are distributed to the body via the bloodstream, H_2_ inhalation cannot exert therapeutic effects within ischemic organs when blood flow is blocked by REBOA in the pig model [19]. Ichihara et al. reported an increase in H_2_ concentration in PV blood after oral ingestion of H_2_-dissolved saline [20]. Although oral ingestion may be one strategy to deliver drugs to the intestinal tract, oral ingestion during hemorrhagic shock is difficult, and continuous oral administration is impractical. Given that H_2_ molecules exert their effect without undergoing metabolism into a pharmacologically active form and can be administered directly into the aorta, our H_2_ delivery approach has the possibility to deliver H_2_ to ischemic organs without any metabolic concern during the hemodynamically unstable phase.

This study had some limitations. First, this is a proof-of-concept study to explore the possibility of a new administration route to visceral organs, including the intestines, under the use of REBOA with intra-aortic irrigation, not an evaluation of efficacy nor a pharmacological study to define an appropriate administration rate. In addition, the minimum effective concentration in pigs remains unknown, although a concentration of 0.016 mg/L (1%) has been documented in other animals, including humans [20]. Second, intra-aortic irrigation was performed in combination with pre-existing devices, including a central venous catheter and REBOA. A 2-in-1 REBOA device, which has a drug-administration port downstream of the block balloon, needs to be newly developed for clinical use. As the size of the REBOA device may increase, blood flow, including drug-dissolved saline, to distal organs, such as the kidney or legs, may decrease owing to intraluminal occupation by the device. Third, because this system may require high-dose intra-aortic administration, there is a risk of air embolism, hemodilution problems, pressure-related problems like abdominal compartment syndrome, and altered distal perfusion for the catheter-inserted leg. Therefore, the evaluation for these complications and a fail-safe function must be established before clinical use. Fourth, the sample size is notably small, even for a proof-of-concept study. This was an exploratory design intended to survey feasibility across multiple models. In particular, the rationale for selecting the administration rate is weak. The high irrigation rate used in the full-occlusion setting is unlikely to be directly applicable clinically. Clinically applicable rates and safety (e.g., hemodilution/pressure effects) require future evaluation with replication. Fifth, this study focused on intestinal drug delivery, and the delivery to other organs was not investigated. Further experiments are warranted to evaluate the drug delivery to other ischemic organs, including the liver, kidney, or legs.

## 5. Conclusions

In this proof-of-concept study, we demonstrated the presence of H_2_ molecules in the PV following REBOA use under various scenarios. The elevated H_2_ concentration in the PV indicates that H_2_ was delivered to the intestine. However, this study did not evaluate whether such delivery confers any physiological benefit. Further investigations exploring this H_2_ delivery approach are required to establish its application in clinical settings.

## Figures and Tables

**Table 1:** Changes in hydrogen concentration in the portal vein.

Model 1								
Shock type		Controlled hemorrhagic shock						
REBOA inflation/time		Full/Standard time						
Irrigation		Hydrogen dissolved saline						
Time	min	Before	5	15	30	45	60	
PV	mg/L (ppm *w*/*w*)	0.008	0.224	0.213	0.172	0.224	0.245	
	%	0.475	13.988	13.294	10.737	13.988	15.303	
**Model 2**								
Shock type		Uncontrolled hemorrhagic shock						
REBOA inflation/time		Full/Extended time						
Irrigation		Hydrogen dissolved saline						
Time	min	Before	5	15	30	45	60	90
PV	mg/L (ppm *w*/*w*)	0.001	0.049	0.035	0.022	0.030	0.080	0.075
	%	0.081	3.063	2.194	1.344	1.894	4.975	4.675
**Model 3**								
Shock type		Uncontrolled hemorrhagic shock						
REBOA inflation/time		Partial/Extended time						
Irrigation		Hydrogen dissolved saline						
Time	min	Before	5	15	30	45	60	90
PV	mg/L (ppm *w*/*w*)	0.003	0.000	0.018	0.020	NA	0.019	0.024
	%	0.213	0.013	1.113	1.263	NA	1.200	1.469
**Model 4**								
Shock type		Controlled hemorrhagic shock						
REBOA inflation/time		Full/Standard time						
Irrigation		Normal saline						
Time	min	Before	5	15	30	45	60	
PV	mg/L (ppm *w*/*w*)	0.000	0.002	0.002	0.002	0.003	0.003	
	%	0.006	0.150	0.138	0.138	0.175	0.188	

PV, portal vein; REBOA, resuscitative endovascular balloon occlusion of the aorta. NA, not available; the PV blood sample at 45 min in Model 3 could not be obtained due to a transient catheter-related technical issue. Each model was performed once (*n* = 1).

## Data Availability

The datasets generated and/or analyzed during the current study are available from the corresponding author upon request.

## Abbreviations

The following abbreviations are used in this manuscript:

CA	Carotid artery
FA	Femoral artery
H_2_	Hydrogen
HR	Heart rate
HS	Hemorrhagic shock
MAP	Mean arterial pressure
REBOA	Resuscitative endovascular balloon occlusion of the aorta
